# miR-370-3p Is a Therapeutic Tool in Anti-glioblastoma Therapy but Is Not an Intratumoral or Cell-free Circulating Biomarker

**DOI:** 10.1016/j.omtn.2018.09.007

**Published:** 2018-09-13

**Authors:** Arulraj Nadaradjane, Joséphine Briand, Gwenola Bougras-Cartron, Valentine Disdero, François M. Vallette, Jean-Sébastien Frenel, Pierre-François Cartron

**Affiliations:** 1 Equipe Apoptose & Progression Tumorale, Centre de Recherche en Cancérologie et Immunologie Nantes Angers (CRCINA), INSERM U1232, Nantes, France; 2 Faculté de Médecine, Université de Nantes, Nantes, France; 3 LaBCT, Institut de Cancérologie de l’Ouest, Saint Herblain, France; 4 Cancéropole Grand-Ouest, réseau Epigénétique (RepiCGO), Université de Nantes, Nantes, France; 5 EpiSAVMEN Consortium (Région Pays de la Loire), Université de Nantes, Nantes, France; 6 LabEX IGO, Université de Nantes, Nantes, France; 7 Department of Medical Oncology, Institut de Cancérologie de l’Ouest site René Gauducheau, Saint Herblain, France

**Keywords:** GBM, miRNA, temozolomide

## Abstract

In the last decade, microRNAs (miRs) have been described as biomarkers and therapeutic agents. Based on this finding, our aim here is to know if (1) miRNA-370-3p can be used as a biomarker associated with a favorable survival and if (2) miRNA-370-3p can be used as a therapeutic tool that increases the efficiency of standard anti-GBM treatment. A first approach using the data available on the “Prognostic miRNA Database” indicated that the expression level of miRNA-370-3p in GBM (T-miR-370-3p) is not associated with a prognosis value for survival. A second approach quantifying the expression level of cell-free circulating miRNA-370-3p (cfc-miR-370-3p) also indicated that cfc-miR-370-3p is not associated with a prognosis value for survival. To investigate whether miR-370-3p can be used *in vivo* to increase the anti-GBM effect of TMZ, we then used the model of LN18-induced GBMs in mice. Our data indicated that the miRNA-370-3p/TMZ treatment was two times more efficient than the TMZ treatment for decreasing the tumor volume. In addition, our study correlated the decrease of tumor volume induced by the miRNA-370-3p/TMZ treatment with the decrease in FOXM1 and MGMT (i.e., two targets of miR-370-3p).

Our data thus support the idea that miR-370-3p could be used as therapeutic tool for anti-glioblastoma therapy, but not as a biomarker.

## Introduction

With an incidence of 2–3 per 100,000 people in Europe and the United States, glioblastoma multiforme (GBM) accounts for 12%–15% of all intracranial tumors and is the most deadly malignant primary brain tumors in adults (http://braintumor.org/). The standard treatment for GBM is based on surgery followed by combined radiation and chemotherapy with temozolomide. Despite these treatments, overall survival (≈15 months) and the 5-year survival rate (≈4%) remain very low, and GBM treatment is in a situation of unmet medical need. To remedy this, intense research into the biology and treatment of GBM has been carried out in the last few decades. One of the most promising strategies provided by this research^1^, ^2^, ^3^, ^4^, ^5^, ^6^ is the use of microRNA (miRNA).^7^, ^8^, ^9^

miRNAs are small (18–25 nt) endogenous non-coding mRNA. These molecules have the ability to inhibit gene expression by binding to target mRNA, thereby promoting a process of translational silencing and/or mRNA degradation. In a clinical setting, there are two advantages to miRNAs: miRNAs can be used as a therapeutic agent or a biomarker.

The literature usually mentions the clinical trial (ClinicalTrials.gov: NCT01829971) using a liposomal formulation of an miRNA-34 mimic (MRX34) as an example. To our knowledge, no clinical trials using therapeutic miRNAs have been reported for GBM, despite robust data supporting a potential therapeutic role for several miRNAs in the treatment of GBM. Of these miRNAs,^10^ there is miRNA-370-3p. Gao et al.^11^ report that the upregulation of miRNA-370-3p restores the sensitivity of GBM cell lines to temozolomide by influencing O-6-methylguanine-DNA methyltransferase (MGMT) expression.

Concerning the biomarker role played by miRNAs in GBM, the literature reports one clinical trial (ClinicalTrials.gov: NCT01849952) evaluating the expression levels of miRNA-10b, as this miRNA regulates the invasion, angiogenecity, and apoptosis of GBM cells.^12^

Based on these findings, we investigated here whether (1) miRNA-370-3p can be used as an intratumoral and/or cell-free circulating biomarker associated with favorable survival and (2) whether miRNA-370-3p can be used as a therapeutic tool that increases the efficiency of the standard anti-GBM treatment.

## Results

### The Intratumoral Expression Level of miR-370-3p Is Not a Biomarker in GBM Patients

**Figure 1:**
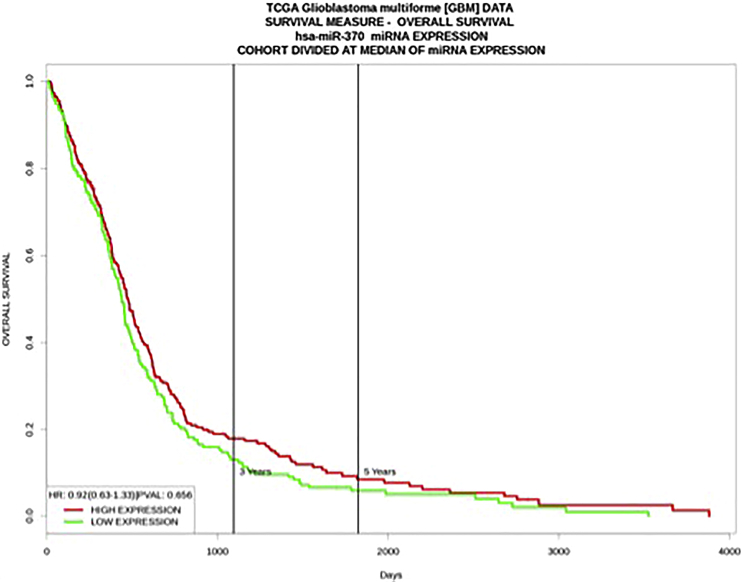
The Tumor Expression Level of miRNA-370-3p Is Not Associated with a Prognosis Value Kaplan-Meier curves illustrate the overall survival of 471 GBM patients divided into two subgroups based on their median value of miRNA-370 expression. Patients whose miRNA-370 expression was greater than the median value are in red (median survival [days] = 476). Patients whose miRNA-370 expression was less than or equal to the median value are in green (median survival [days] = 441).

To investigate whether miR-370-3p can be used as a biomarker associated with favorable survival, we first analyzed the data available in the Prognostic miRNA Database (http://xvm145.jefferson.edu/progmir/index.php). We observed that the expression level of miRNA-370-3p in GBM (T-miR-370-3p) is not associated with either a prognosis value for survival or for overall survival (OS) (Figure 1A).

### The Cell-free Circulating Expression Level of miR-370-3p Is Not a Biomarker in GBM Patients

**Figure 2:**
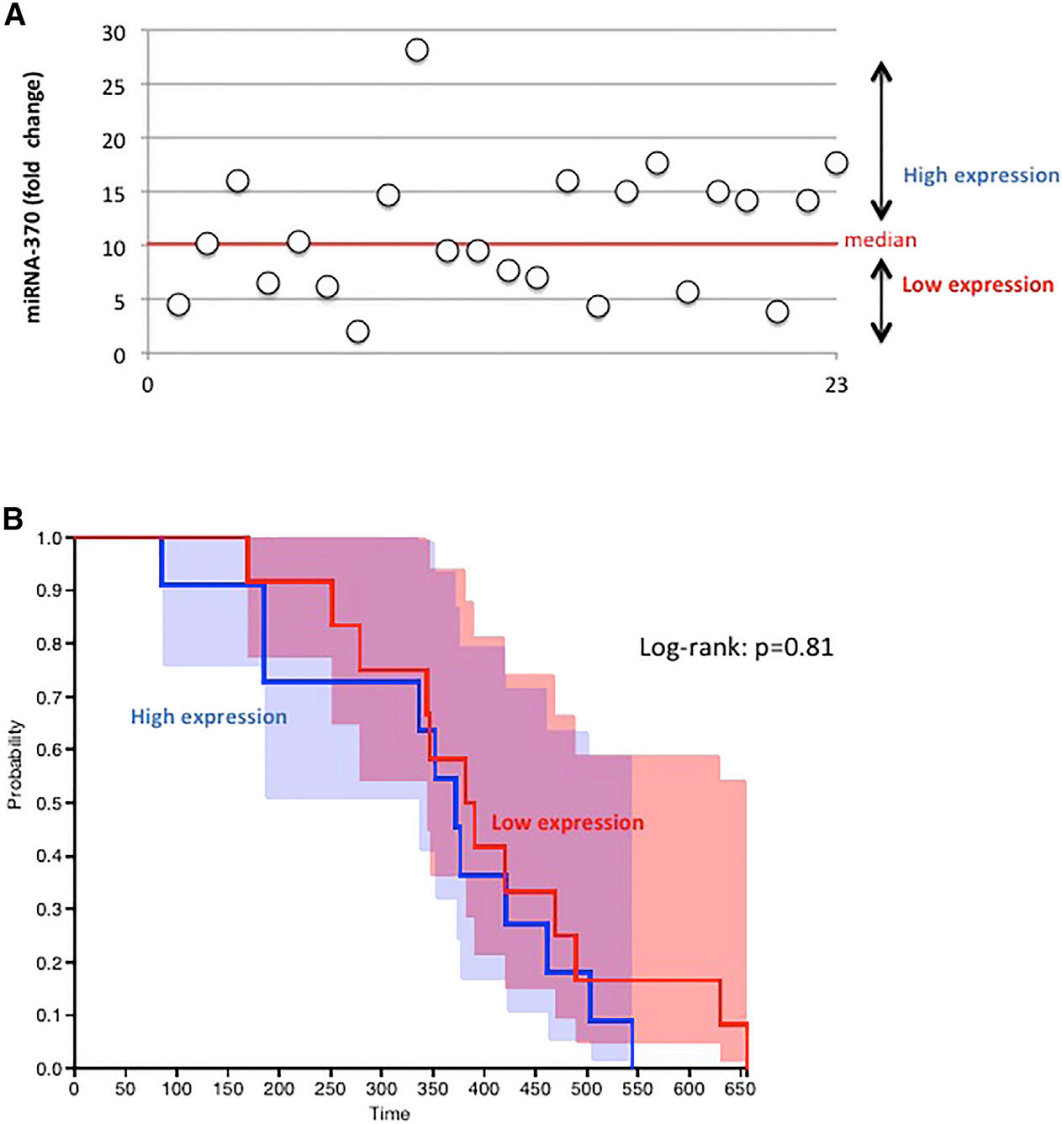
Cell-free Circulating miRNA-370 Is Not Associated with a Prognosis Value (A) Expression of cell-free circulating (cfc) miRNA-370-3p was estimated by qRT-PCR for each patient (n = 23). Each open circle symbolizes one patient. (B) Kaplan-Meier curves illustrate the overall survival of 23 GBM patients divided in two subgroups based on their median value of cfc-miRNA-370 expression. Patients whose cfc-miRNA-370 expression was greater than the median value are in blue (n = 11, median survival [days] = 372). Patients whose cfc-miRNA-370 expression was less than or equal to the median value are in green (n = 12, median survival [days] = 386).

In a second approach, we considered the cell-free circulating expression level of miR-370-3p (cfc-miR-370-3p). The cfc-miR-370-3p expression level was analyzed from the plasma of 23 GBM patients treated at the Institut de Cancérologie de l’Ouest. The main characteristics of these patients are summarized in Table 1. qRT-qPCRs were performed to estimate the expression level of cfc-miR-370-3p (Figure 2A). Our cohort of 23 samples was then divided into two subgroups using the median value as threshold. Survival curves were visualized in a Kaplan-Meier plot. A log-rank test indicated a lack of difference between the overall survival of GBM patients with a high level of cfc-miR-370-3p and those with a low level of cfc-miR-370-3p (Figure 2B).

**Table 1:** Characteristics of the GBM Patients

Characteristics	Patients (n = 23)	Log-Rank Test
Age (years) median [min;max]	61 [36;80]	p = 0.42

**Gender (n)**		

Male	11	p = 0.28
Female	12	

**Survival Time (Days)**		

Median [min;max]	376 [85;656]	

**Extent of Surgery**		

Biopsy or partial resection	7	p = 0.37
Complete resection	16

### miR-370-3p Downregulates the MGMT Expression and Increases the Temozolimide-Induced Cell Death

To investigate whether miR-370-3p can be used as a therapeutic tool increasing the efficacy of the standard anti-GBM treatment, we first investigated whether miRNA-370-3p affects the MGMT expression level and increases the sensitivity of GBM cells to temozolomide, as previously reported.

**Figure 3:**
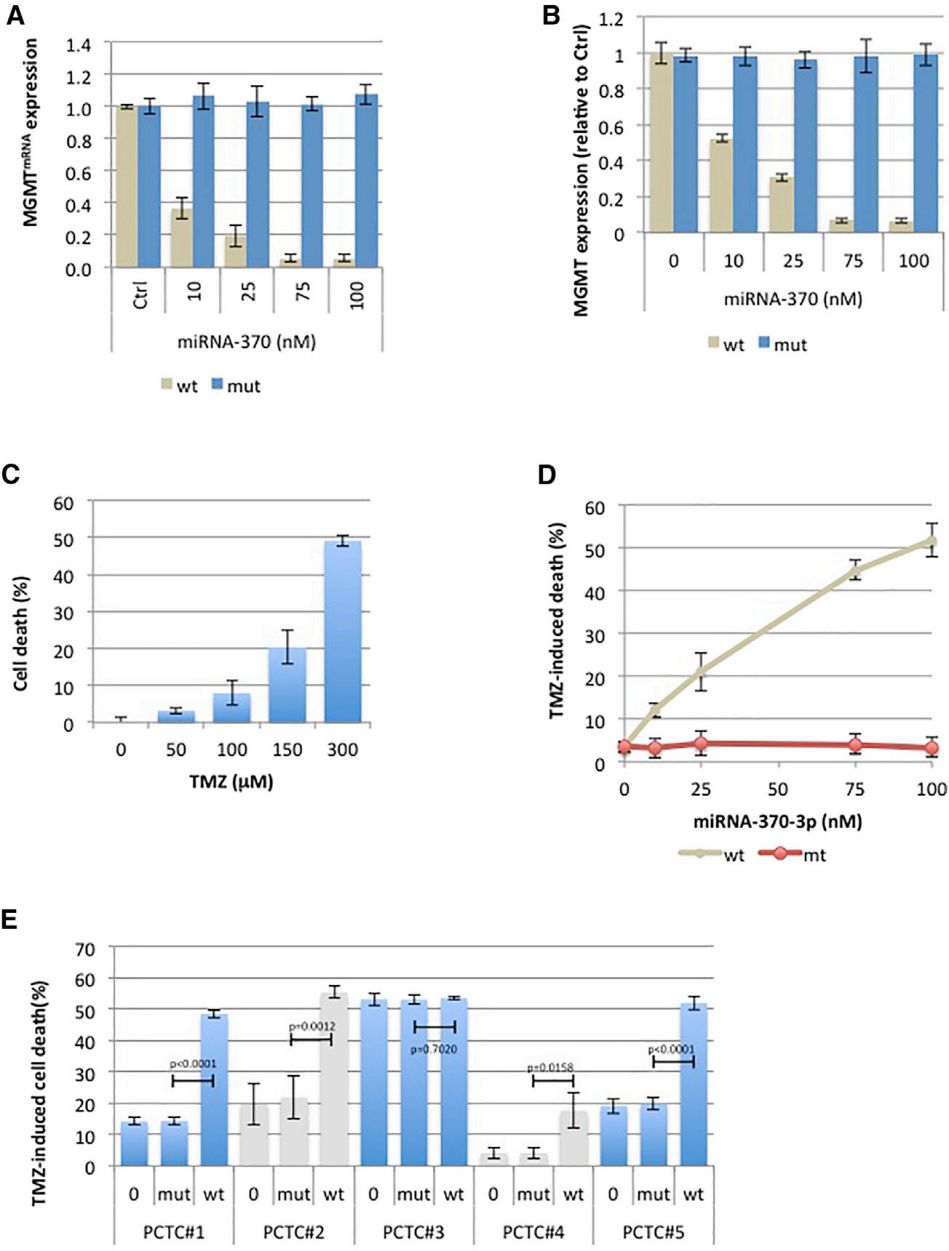
miRNA-370-3p Downregulates the Expression of MGMT and MGMT^mRNA^ and Increases Sensitivity to TMZ *In Cellulo* (A and B) qRT-PCR (A) and ELISA (B) indicate that miRNA-370-3p decreases MGMT expression at the mRNA and protein levels, respectively. Mimetic wild-type (gccugcugggguggaaccuggu) or mutated (gAAugcAAggguggaaAAuggu) miR-370-3p were transfected using HiPerFect transfection reagent (QIAGEN, France) according to the manufacturer’s instructions. (C) Response of the LN18 cell line to treatment with TMZ for 72 hr was assessed by cytotoxicity assay (Abcam, ab197010, France). (D) The impact of dose-escalation miRNA-370 on TMZ (50 μM)-induced cell death was estimated by cytotoxicity assay (Abcam, ab197010, France). (E) The impact of TMZ (50 μM)-induced miR-370-3p-mediated cell death was estimated by cytotoxicity assay (Abcam, ab197010, France) on a panel of five distinct primary-cultured tumor cells (PCTC). Mimetic wild-type or mutated miR-370-3p (25 nM) were transfected using HiPerFect transfection reagent (QIAGEN, France) according to the manufacturer’s instructions. A t test (GraphPad software) compares the mean ± SD of indicated values. Histograms represent average ± SD of 3 independent experiments of the considered parameters.

To perform this investigation, we used LN18 cell lines, i.e., a cell line expressing MGMT. As expected, a first set of experimentations confirmed that miR-370-3p affected the MGMT^mRNA^ and MGMT expression levels in a dose-dependent manner (Figures 3A and 3B).

A second set of experimentations indicated that miR-370-3p increased temozolomide (TMZ)-induced cell death on LN18 cells in a dose-dependent manner (Figures 3C and 3D).

To determine whether the miR-370-3p-mediated increase of TMZ-induced cell death is not a phenomenon specifically associated with the consideration of LN18 cells, we then asked whether miR-370-3p could increase TMZ-induced cell death on a panel of primary-cultured tumor cells (PCTCs). Figure 3E shows that miR-370-3p increased TMZ-induced cell death in 4/5 PCTCs. Interestingly, we noted that the PCTCs with no gain of miR-370-3p-mediated sensitivity to TMZ-induced cell death is a PCTC with a high level of TMZ-induced cell death. Our data thus suggest that the miR-370-3p-mediated increase of TMZ-induced cell death is a general phenomenon, not restricted to LN18 cells.

### miR-370-3p Also Targets FOXM1, but Not FOXO1 or TGFβ−ΡII

**Figure 4:**
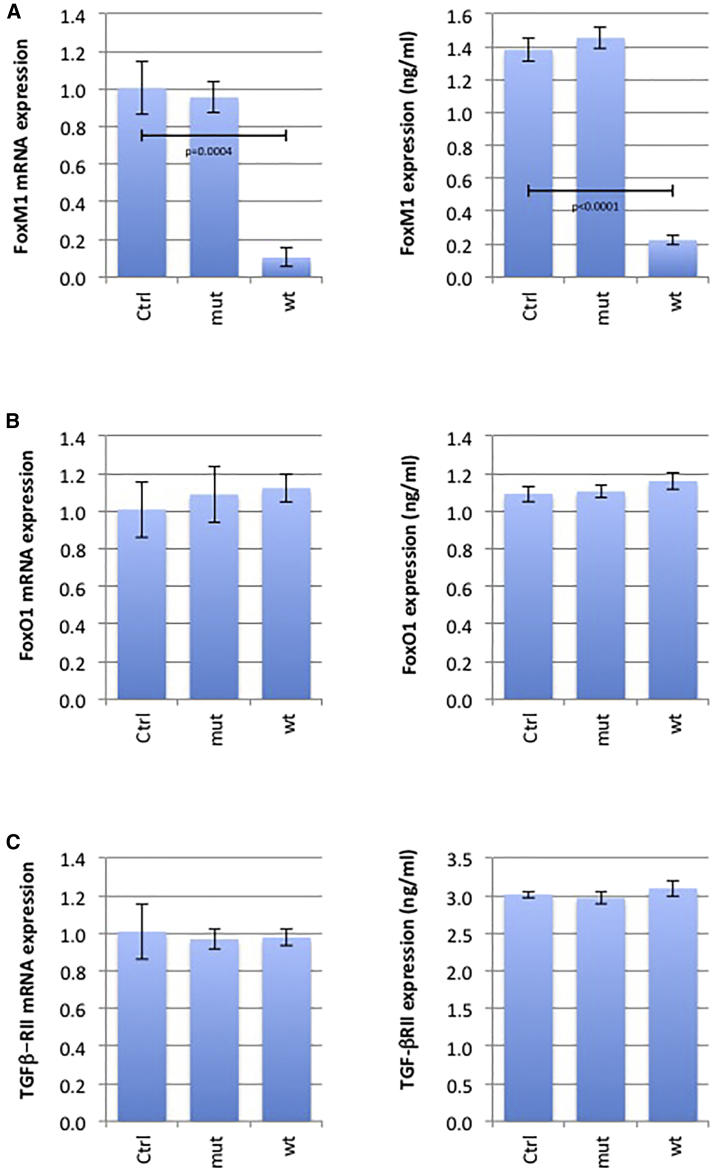
miR-370-3p Also Targets FOXM1, but Not FOXO1 and TGFβ-RII LN18 cells were untreated or treated with mimetic miR-370-3p or mutated mimetic miR-370-3p. qRT-PCR and ELISA analyzed the expression level of FOXM1 (A), FOXO1 (B), and TGFβ-RII (C) at mRNA and protein levels, respectively. Mimetic wild-type or mutated miR-370-3p (25 nM) were transfected using HiPerFect transfection reagent (QIAGEN, France) according to the manufacturer’s instructions. A t test (GraphPad software) compares the mean ± SD of indicated values. Histograms represent average ± SD of 3 independent experiments of the considered parameters.

As miRNA has multiple targets, we considered three other targets for miR-370: forkhead box protein M1 (FOXM1), FOXO1, and transforming growth factor β (TGFβ)-RII (Table 2). ELISA and qRT-PCR indicated that only FOXM1 expression was affected at the mRNA and protein level by miR-370-3p (Figure 4).

**Table 2:** Prognosis Value of miRNA-370-3p Targets

	Validation Methods			Prognosis Value	
	Reporter Assay	Western Blot	qPCR	Log-Rank Test	Expression Associated with a Favorable Prognosis
TGFβ-RII	✔	✔	✔	p = 0.336	–
FOXO1	✔	✔	✔	p = 0.248	–
FOXM1	✔	✔	✔	p = 0.0219	low

Here, the targets for miRNA-370-3p considered to be identified by the miRTarBase website. We considered targets validated by three strong pieces of evidence, i.e., targets validated by the use of three different methods (reporter assay, western blot, qPCR) commonly used to analyze the effect of miRNA on its targets (according to miRTarBase website criteria). Prognosis values were calculated using the Betastasis website and the REMBRANDT (repository for molecular brain neoplasia data) bioinformatics knowledgebase available for GBM (http://www.betastasis.com/glioma/rembrandt/kaplan_meier_survival_curve).

### miR-370-3p Increases Temozolomide Sensitivity *In Vivo*

**Figure 5:**
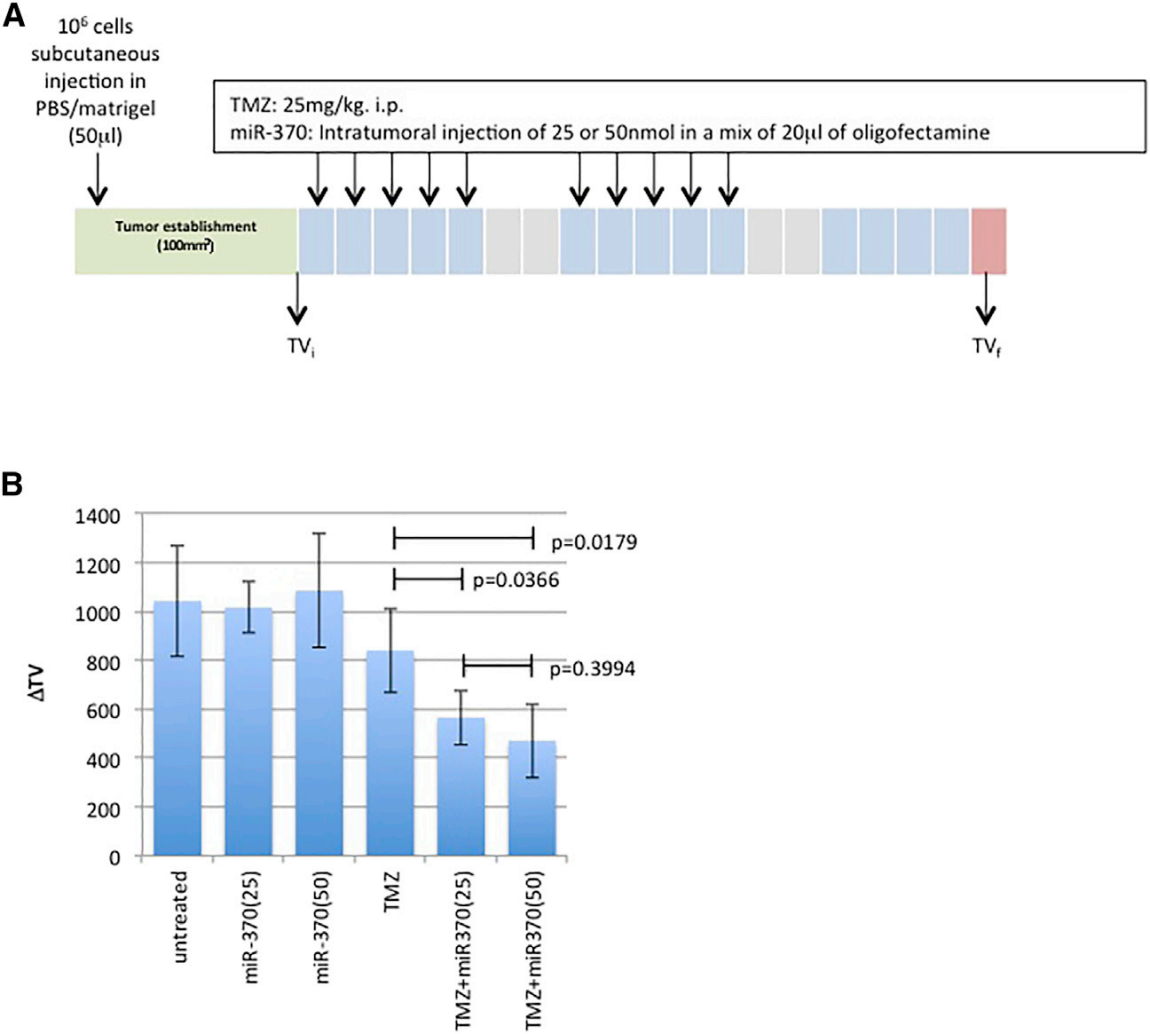
miRNA-370 Increases the Sensitivity of TMZ *In Vivo* (A) Schematic representation of the protocol used to treat mice. BALB/c nude mice (female, 5–6 weeks old) with LN18 tumors were separated into six treatment groups (four mice per group); None, miR-370(25), miR-370(50), TMZ, TMZ + miR-370(25), and TMZ + miR-370(50). TMZ (25 mg/kg) and/or miR-370-3p were administered intraperitoneally (i.p.) and intratumorally (i.t.) on days 1, 2, 3, 4, and 5 of each week for 2 weeks. Tumor volumes were measured *in situ* with digital calipers at the indicated time in order to evaluate initial tumor volume (TVi) and final tumor volume (TVf). (B) The growth in tumor volume (ΔTV) was calculated as follows: ΔTV = TVf − TVi. Each bar represents mean ± SD calculated from four mice. A t test (GraphPad software) compares the mean ± SD of indicated values. Histograms represent average ± SD of 3 independent experiments of the considered parameters.

We investigated whether miR-370-3p can be used *in vivo* to increase the anti-GBM effect of TMZ. For this purpose, LN18-induced GBMs were generated by xenograft in mice. When the volume of the LN18-induced GBMs was close to 100 mm^3^, four mice were randomly untreated or treated with TMZ and/or miR-370-3p at two concentrations (Figure 5A). By comparing the effect of the TMZ treatment with the effect of the TMZ/miR-370-3p treatment, we could see clearly that the latter treatment was more efficient than the TMZ treatment (Figures 5B and 5C).

### The Decrease in Temozolomide/miR-370-3p-Induced Tumor Volume Correlates with the Decrease in Expression of MGMT and FOXO1, Two of the Targets of miR-370-3p

**Figure 6:**
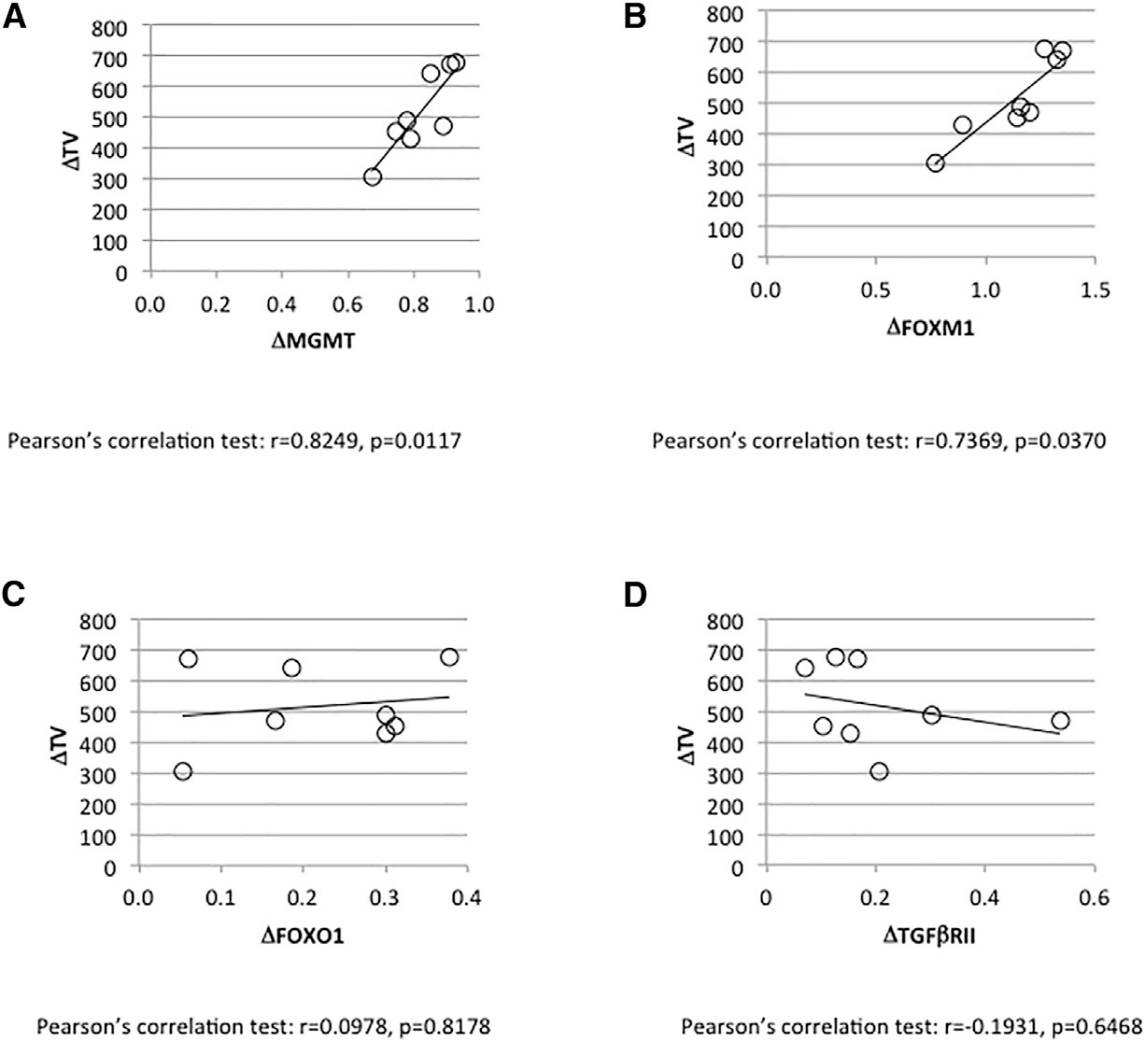
Study of the Correlation between the TMZ/miR-370-3p-Induced Decrease in Tumor Volume and the Modulation in Expression of Four miRNA-370-3p Targets MGMT (A), FOXM1 (B), FOXO1 (C), and TGFβ-RII (D).

We next analyzed the putative correlation between the TMZ/miR-370-induced reduction in tumor volume and the TMZ/miR-370-induced reduction in MGMT expression. This last parameter was estimated by calculating the difference in MGMT expression at protein levels between the MGMT expression seen in LN18 cells and in resected tumors. ELISA was used to do this. By considering the eight tumors previously treated with TMZ/miR-370-3p, we noted a correlation between the miR-370-induced reduction in tumor volume and the miR-370-induced reduction in MGMT expression (Figure 6A).

Interestingly, we noted that the miR-370-induced reduction in tumor volume was also correlated with the miR-370-induced reduction in FOXM1 expression (Figure 6B). However, the miR-370-induced reduction in tumor volume was not correlated with the miR-370-induced reduction in FOXO1 or TGFβ-RII expressions (Figure 6C).

### Study of the Longitudinal Expression of miR-370-3p during Standard Anti-GBM Treatment

**Figure 7:**
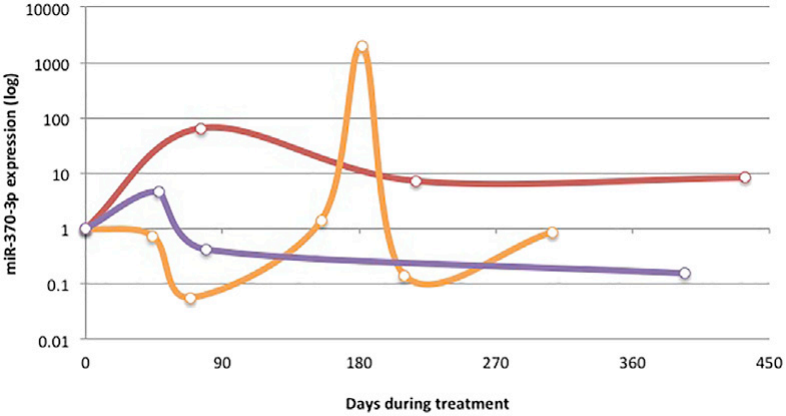
Study of the Longitudinal Expression of miR-370-3p during the Standard Anti-GBM Treatment The graph illustrates the changes in miR-370-3p expression during the standard anti-GBM therapy received by seven patients. Each curve symbolizes one patient. Each date of sampling is symbolized by a circle on a curve. T = 0 represents the first day of standard anti-GBM treatment received by a considered patient.

Our data paradoxically indicated that expression of miR-370-3p is not associated with a prognosis value of response to standard anti-GBM, whereas the addition of this miR increased sensitivity to the standard anti-GBM treatment in *in cellulo* and *in vivo* models of GBM. Based on this point, we postulated that miR-370-3p could be dynamically modulated (up- and downregulated) during the administration of standard anti-GBM treatment. To investigate this point, we analyzed the expression level of cfc-miR-370-3p in longitudinal blood samples of three GBM patients treated with the standard anti-GBM treatment. Figure 7A shows that miR-370-3p expression was dynamic during standard treatment of GBM patients. This last fact suggests that the consideration of miR-370-3p expression as a biomarker requires the realization of a longitudinal study.

## Discussion

The aim of an extensive amount of research is to identify miRNA as a biomarker and/or therapeutic agent. Our article is also a part of this research axis as it investigated the biomarker and/or therapeutic agent role that miRNA-370-3p might play in GBM.

From our data, we can conclude that miR-370-3p is a therapeutic tool in anti-glioblastoma therapy but not an “in initial tumor” or initial cell-free circulating biomarker. The absence of a biomarker value for miRNA-370-3p in initial GBM or blood is supported by consideration of 471 samples in the data available in the Prognostic miRNA Database and consideration of 23 samples in our cohort of samples. To data, Hayes et al.^13^ and Li et al.^14^ have reported that miRNA-370-3p expression is protective in neural subtype of GBM. Gao et al.^11^ reported that miRNA-370-3p has significantly lower expression in GBM tissue compared to paired non-cancerous tissue, and this independently of the GBM subtypes. However, these studies did not investigate the prognosis value of miRNA-370-3p expression in GBM. In tumors other than GBM, miRNA-370-3p expression had a prognosis value in hepatocellular carcinoma^15^ and in pediatric acute myeloid leukemia^16^ for example, but also in non-cancer diseases such as coronary artery disease^17^ and hyperlipidemia.^18^ The longitudinal analysis of miR-370-3p from blood collected during tandard anti-GBM therapy received by patients revealed a relationship between the miR-370-3p expression changes and the time of patient survival before relapse: the longer miR-370-3p is overexpressed, the longer the patient survival before relapse is long. This observation is consistent with the fact that our work identifies miRNA-370-3p as a therapeutic agent potentiating the anti-GBM effect of temozolomide on GBM cells. By showing that 50% of cell death is induced by 300 μM of TMZ or by 50 μM of TMZ + miR-370-3p, our data introduce the idea that using miR-370-3p could make it possible to reduce the quantity of temozolomide and by extension reduce the secondary effects associated with using of temozolomide. In addition, longitudinal miR-370-3p monitoring in blood during the anti-GBM treatment could make it possible to observe that miR-370-3p expression is dynamic during standard anti-GBM treatment. Consequently, our study needs to include more patients to determine whether the time period characterized by elevated miR-370-3p expression is associated with a favorable prognosis of response.

Gao et al.^11^ have reported that miRNA-370-3p sensitizes the response of GBM cells to temozolomide via the downregulation of MGMT. Our study also supports this data and is more advanced by providing *in vivo* results. For the first time in GBM, our study shows that miRNA-370-3p potentiates the anti-GBM effect of temozolomide in *in vivo* models of xenograft GBM. However, this effect has already been described for tumors other than GBM. For example, Liu et al.^19^ report that miRNA-370 inhibits the growth and metastasis of lung cancer. The tumor-suppressive function of miRNA-370 is also reported in both acute myeloid leukemia^20^ and laryngeal squamous cell carcinoma.^21^ While these results and our data support the idea of using miR-370 as anti-cancer agents, several other publications report that miR-370-3p plays an oncogenic role. Thus, Lo et al.^22^ show that overexpression of miRNA-370-3p contributes to gastric carcinoma, and Wei and Ma^23^ report that miR-370-3p acts as an oncogene in melanoma.

The tumor suppressor or oncogene roles played by miRNA-370-3p could be due to its targets. When miRNA mainly represses the expression of oncogenes, it is considered to be a tumor suppressor, while an miRNA that mainly represses the expression of tumor-suppressor genes is considered to be an oncogene (also named oncomiR). With regard to miR-370, Zhang et al.^20^ demonstrated that the tumor-suppressive role of this miRNA in acute myeloid leukemia is associated with the targeting of FOXM1, and FOXM1 is mainly considered an oncogene in the literature.^24^ We and Gao et al.^11^ have associated the tumor-suppressive role of miRNA-370 in GBM with the targeting of MGMT. On the contrary, Lo et al.^22^ have associated the progression of gastric carcinoma with the miRNA-370-3p-induced downregulation of TGFβ-RII, and TGFβ-RII is associated with a tumor-suppressive pathway.^25^ In our *in vivo* study, miRNA-370-3p plays a tumor-suppressive role, and this role is associated with the MGMT and FOXM1 downexpressions, while the expressions of TGFβ-RII and FOXO1 remain unchanged.

In conclusion, our study supports the idea of using miR-370-3p in an miR-based treatment of GBM.

## Materials and Methods

### Plasma Samples

Plasma was collected from GBM patients treated at the Institut de Cancérologie de l'Ouest (ICO, http://www.ico-cancer.fr). In accordance with the regulations, all subjects signed a specific informed consent form for this biocollection, approved by an Ethics Committee (CPP OUEST IV, no. 18/16), the French State Department for National Education, Higher Education and Research (Ministère de l’Education Nationale, de l’Enseignement Supérieur et de la Recherche, no. DC-2015-2457) and the CNIL (compliance commitment to MR 001).

### Cell Culture Conditions

LN18 cells were cultured in high-glucose DMEM with addition of 10% of heat-inactivated fetal bovine serum, streptomycin (100 μg/mL), penicillin (100 U/mL), and 2 mmol/L L-glutamine. Cells were cultivated in a 5% CO_2_ incubator at a temperature of 37°C. Cells reaching sub-confluency were detached from the culture dishes using 0.05% trypsin 0.02% EDTA in calcium-free PBS and counted in a Scepter cell counter (Millipore).

### PCTCs

Fresh brain-tumor tissue obtained from the neurosurgery department of the Laennec Hospital (Nantes/Saint-Herblain, France) was collected and processed within 30 min after resection. The clinical protocol was approved by the French laws of ethics with informed consent obtained from all subjects. The primary cultured tumor cells were obtained after mechanical dissociation using the technique previously described.^26^ In brief, tumor tissue was cut into pieces of 1–5 mm^3^ and plated in a 60-mm^2^ tissue culture dish with DMEM with 10% FBS and antibiotics. Additionally and in parallel, minced pieces of tumor were incubated with 200 U/mL collagenase I (Sigma, France) and 500 U/mL DNaseI (Sigma, France) in PBS for 1 hr at 37°C with vigorous constant agitation. The single-cell suspension was filtered through a 70-mm cell strainer (BD Falcon, France), washed with PBS, and suspended in DMEM-10% FBS. Cell cultures were subsequently split 1:2 when confluent and experiments were carried out before passages 3–5. During this period, cells were maintained at 37°C in a humidified atmosphere containing 5% CO_2_ air.

### miRNA Extraction and qRT-PCR

A QIAcube automate and miRNEasy Serum/Plasma Kit (QIAGEN, France) were used to isolate circulating miRNA. miScript II RT, miScript SYBR Green PCR kits, and miScript Primer Assays (QIAGEN, France) were used to perform the qRT-PCR on the Rotor-Gene Q (QIAGEN, France). Quantification and purity were analyzed using Qubit (Thermo, France) and Agilent 2100 (Agilent Small RNA kit) according to the manufacturer’s instructions, respectively.

### *In Vivo* Experiments

Cultured LN18 cells were harvested by trypsinization, washed and resuspended in saline buffer. Cell suspensions were injected subcutaneously (s.c.) into the flank of 7- to 8-week-old mice (Janvier, France).

Tumor volume based on caliper measurements was calculated using the modified ellipsoidal formula (tumor volume = 1/2(length × width^2^)) according to previous data. At the end of the 21-day observation period, the mice with xenograft tumors were euthanized, and the tumor tissues were removed for analysis.

The experimental procedures using animals were in accordance with the guidelines of Institutional Animal Care and the French National Committee of Ethics. In addition, all experiments were conducted according to the Regulations for Animal Experimentation at the Plate-forme Animalerie in the Institut de Recherche en Santé de l'Université de Nantes (IRS-UN) and approved by the French National Committee of Ethics.

### mRNA Extraction and qRT-PCR

A QIAcube automate and RNeasy Mini QIAcube Kit (QIAGEN, France) were used to isolate the mRNA. QuantiTect Reverse Transcription, QuantiFast SYBR Green PCR Kits, and QuantiTect Primer Assays (QIAGEN, France) were used to perform the qRT-PCR on the Rotor-Gene Q (QIAGEN, France).

### Protein Analysis: ELISA

Protein extracts were obtained using RIPA Lysis and Extraction Buffer (Thermo Scientific, France) in accordance with the manufacturer’s instructions. ELISAs were performed according to the manufacturer’s instructions (MyBiosource, USA).

## Author Contributions

P.-F.C. designed and coordinated the project. A.N., J.B., and P.-F.C. performed all the experiments. G.B.-C., V.D., and J.S.-F. coordinated the obtaining and use of patient samples. G.B.-C., F.M.V., J.-S.F., and P.-F.C. interpreted and discussed the data. P.-F.C. wrote the first version of the manuscript and all authors reviewed and approved it.

## Conflicts of Interest

The authors have no conflicts of interest.
